# Unusual Distention of the Cavity of the Cervix Uteri, with Spasmodic Contraction of the Internal Os, in a Case of Miscarriage

**Published:** 1878-11

**Authors:** Norman Bridge


### UNUSUAL DISTENSION OF THE CAVITY OF THE CERVIX UTERI, WITH SPASMODIC CONTRACTION OF THE INTERNAL OS, IN A CASE OF MISCARRIAGE.

By Norman Bridge, M. D.

(*Read, in abstract, before the West Chicago Medical Society, Sept.* 23, 1878.)

Mrs. X., æt. 36 years, a healthy American woman, who had borne three children at term—the youngest being 4 years old and the oldest 18—was threatened with miscarriage at the end of the fourth month. She had had some irregular pain in the back and had been losing considerable blood for three days; so that she was very pale and so weak she was obliged to keep in bed. The uterus was found to be of the globular shape and size characteristic of the period of gestation existing; the cervix was of normal shape, projecting from the mass of the uterus half or three-quarters of an inch, and the os was closed. No blood was being lost at the time of the examination—at least none of any consequence—and no interrupted pains were occurring. The recumbent posture, small doses of morphine and ipecac, as required by pain, and an occasional ten-grain dose of gallic acid, were ordered.

For two days the indications were that an abortion would be averted. There was no pain or loss of blood and the shape and prominence of the cervix did not change. Then gradually the cervical projection disappeared and the os became patulous, and there was some loss of blood. At the end of five and a half days from the first visit an examination revealed no projection of the cervix beyond the uterine globe; the os was dilated to the size of a silver half-dollar. The patient had been discharging blood in considerable quantity for twelve hours. Two teaspoonfuls of Squibb's fl. ex. ergot had been taken in divided doses since the flowing began. Not more than six pains at all like labor pains had been experienced during this time. The flowing had ceased. An attempt was now made to further dilate the os with the fingers and assist the uterus to expel its contents. Patient attempts at dilatation availed little—the border of the os becoming hard like a cord on every effort to stretch it, however gently the pressure was applied. The opening was already sufficient for the exit of clots of considerable size, and these bodies were hooked and drawn out of the cavity by the finger, until the bulk of them became surprising if not alarming.

After extracting all the clots that could be reached, and what I think it no exaggeration to say would amount to a pint and a half in quantity, effort was made to reach some part of the fœtus. But instead of grasping the fœtus, the finger, as it was pushed up, only struck the hard border of another opening to a cavity beyond. This opening was recognized as the internal os uteri and the cavity as that of the body of the uterus.

All these clots, then, had been contained in the cavity of an elongated cervix uteri. I am positive that during the extraction of the clots none of them came down from the uterus, as there was no movement of the jelly-like body of blood from above, and furthermore most of the masses were too large to pass unbroken through the internal opening.

The distance from the external to the internal os was about four inches—judging from measurements made with the finger.

The internal os was dilated less than the external; it was exceedingly hard and unyielding and presented a sharp, hard border. The walls of the body of the cervix were quite soft and distensible, and it was not surprising that they had been able to enclose such a mass of blood clots.

It was the work of nearly or quite half an hour to dilate the internal os with the fingers, and grasp and extract the fœtus and placenta. The feet of the fœtus were first secured, and brought down with little difficulty, and the body drawn through the opening, when the internal os immediately contracted about the neck, and refused to allow the head to pass. The long delay was caused by the difficulty encountered in delivering the head, and no one who has not had a similar experience can appreciate how great the obstacle was to the execution of this maneuver, generally so simple and easy. The head was of course small and elastic—easily moulded to almost any shape; but the internal os closed to a diameter much less than that of this body, and presented a border or ring that was so sharp and firm as to give an impression to the fingers as though a string of catgut, imbedded beneath the mucous membrane, so as to encircle the cavity at this point, had been drawn together to this small diameter, and tightly tied.

Little difficulty was found in extracting the placenta. The uterus being emptied, contracted firmly. In this contraction the cervix was involved to a very slight degree only, for to the touch it still seemed like a soft, fleshy bag of considerable size, empty and collapsed. The body of the uterus, in distinct contrast, was quite hard.

This case, it appears to me, is a peculiar and interesting one, in the fact that the cervix uteri had undergone an unusual elongation, and a distension of its walls that transformed it into a cavity as large, or nearly as large, as the cavity of the uterine body; that this cavity was guarded by a positive contraction of the external, and a very much more pronounced and extensive one of the internal os; that the latter, by contracting upon the neck of the fœtus, interposed a serious obstacle to the extraction of the head after the body had been successfully delivered; and that all this should have occurred in a patient not advanced to the fifth month of pregnancy.

It is now tolerably well demonstrated that the cervix uteri may become, during parturition, elongated and distended until it is capable of accommodating a considerable portion of the mass of the child; and in cases where this occurs—whether as a cause, or consequence, or neither—that the internal os may be contracted with such force as to make natural delivery impossible.

The case here reported shows the possibility of a similar combination of unusual phenomena in miscarriage before half the normal period of gestation has been reached. I am not aware that any case similar to this has heretofore been recorded, although one resembling it in the spasm of the internal os, as well as in the stage of gestation at which it occurred, is quoted at length near the close of this paper. Another case, reported by Dr. Goelet, and presently to be described, bears possibly a still stronger resemblance to the one just detailed.

Dr. Hosmer,* of Massachusetts, has reported—from his own practice and that of others—four instances of women who have, during parturition at term, had the misfortune to have distocia from spasmodic contraction of the internal os. One woman had two labors thus complicated; another three; the rest, one each.

The mothers all died as a consequence of the distocia and the ordeal; two died undelivered.

In each labor the same difficulty presented itself. All the presentations were of the head, and the obstacle to delivery was a sharp contraction of what was evidently the internal os, grasping the child somewhere between the head and hips after the former had passed out of the cavity of the uterus and was contained in the enormously elongated cervix. In all the cases where delivery was accomplished, version had to be resorted to, and this operation was, in some of the cases, an exceedingly difficult one, owing to the force of the contraction of the internal os. In nearly or quite all the cases the forceps were first tried, but in no single instance was it possible to deliver the fœtus head first.

The constriction referred to was, by the attendant in each case, supposed to be in the uterine cavity proper, and it is no wonder it was taken for an hour-glass contraction according to the old descriptions, occurring ante partum. In one case it is described as "midway between the os and fundus;" "a powerful constriction, grasping and holding the pelvis of the child like a gigantic sphincter, whose force, perhaps, was surely to be augmented through the law of reflex action by every attempt that was made to overcome and remove it."

In another case, the observer, in describing the efforts at version, says: "Before the leg could be found the hand reached what, from the rounded outline, was supposed to be the fundus of the

* "A peculiar condition of the cervix uteri which is found in certain cases of distocia."—*Boston Med. and Surg. Journal, March* 21, 1878.

womb. Under the impression that the position of the fœtus might have been mistaken, the hand was partially withdrawn. This question having been settled, the hand was again pushed upwards, and then discovered that what had been at first supposed to be the fundus was really a constriction about the upper third of the uterus, encircling the child and inclosing its hips and lower extremities." "It seemed like an hour-glass contraction, and consisted of a large, firm band, with its inner edge quite sharp." "The upper surface of the constriction seemed flat, and to the touch suggested the edge of a board or shelf." So strong was the constriction that the "hand, now within and beyond it, could not be opened, and the arm was paralyzed and almost useless."

Dr. D. S. Kellogg,* of New York, has recently reported a case of what he evidently supposed at the time of its occurrence, to be an hour-glass contraction of the uterus, but which, from the description, it can hardly be doubted, was a case identical in character with those reported by Dr. Hosmer. Dr. K. says in his paper: "There was a nearly circular band about one-third of the distance from the internal os uteri to the fundus, which band was higher up behind than in front, and which closed down upon the child like a strong belt of leather."

The constriction was so unyielding and so powerful, that in attempting to dilate it with the patient anæsthetized, "each one [three physicians being in attendance] in turn, until exhausted, gently, but firmly, tried to insinuate his hand into the stricture. After an hour or two we were rewarded by one foot, then presently by the other." After version, and the delivery of the fœtus through the constriction, no obstacle was met to its complete delivery into the world, until the head reached the "internal os uteri," when a slight contraction occurred at this point, but did not last long. Such difficulty was met with in extracting the head from the pelvis, however, that the fœtus had to be decapitated, and the head emptied and crushed. A measurement subsequently showed the antero-posterior diameter of the superior strait, to be less than 3½ inches.

---

*"An interesting and unusual case of obstetrics." By D. S. Kellogg, M. D., etc., *Medical Record*, Aug. 31, 1878.

Were it not for the fact that Dr. K. speaks in two places of the unnatural constriction as being above the *internal* os uteri, no one would regard this case as differing from those reported by Dr. Hosmer. If Dr. K. is certain he demonstrated the existence of both ora—the internal as distinct from the external os, then his case forms a variety different from the others referred to, and this whole subject acquires thereby, a greatly increased interest.

In the absence, however, of a more positive statement on the point alluded to, readers will be inclined to class all these cases as alike in anatomical characteristics.

In 1870 Dr. Gray* reported to the Buffalo Medical Association a case of what he believed to be hour-glass contraction, occuring before the birth of either child or placenta. After referring to the fact that certain authors had denied that hour-glass contraction occurring after the delivery of the child, was produced by anything but the contraction of the internal os—it being claimed that there is no such thing as hour-glass contraction of the body of the uterus—and after expressing his belief in this occurrance according to the old descriptions in the books, he proceeded to give a history of his case.

His patient "had had five or six difficult labors, giving birth to still-born children." She had been in labor 48 hours when the doctor was called to her in consultation. The presentation was of the vertex ; forceps had been used and these failing, craniotomy had been performed before his arrival. Dr. Gray then again tried the forceps, failed and introduced his left hand to turn the child. He seized a foot, but found he could neither bring that down nor withdraw his hand "on account of the constriction of the circular fibers of the muscles of the uterus." "Any effort to remove the hand from the uterine cavity would excite such strong muscular contractions that it became impossible while the hand was closed." "The constricted portion of the uterus appeared to be the size of a rope one inch in diameter, felt as hard as a bone, and at first was mistaken for bone." "Its contractile power was so forcible that the pain inflicted upon the wrist at each uterine

---

* *Buffalo Medical and Surgical Journal*, January, 1871. Half yearly Compend. July, 1871.

contraction, was almost insupportable." The head of the child "was below the rope-like and constricted muscles." He was positive that the uterine contraction "was not that of the os uteri, but existed above it." After the leg was brought down the turning could not be completed "by the strongest traction." But by pressing strongly against the head, forcing it back through the hour-glass contraction and making strong traction by the leg, delivery was finally accomplished. The woman had a mal-formed pelvis, the promontory of the sacrum was unusually prominent and the conjugate diameter was not more than three inches. The operation was made with the aid of anæsthesia. The woman recovered.

Evidently the possibility of an elongation of the cervix that would make it spacious enough to hold the head of the child, had not occurred to the reporter of this case. Without such a possibility in mind any practitioner would probably have arrived at the conclusion Dr. Gray did as to the nature of such a complication. Yet no one reading Dr. Hosmer's paper and this report could doubt—it seems to me—that the same pathological condition existed in all the cases recorded by both. This view is strengthend by the fact that in Dr. Gray's case there was a resemblance to most of the cases of Dr. H., and that of Dr. K., in a deformed pelvis, that had proved an obstruction to delivery, and had presumably caused the death of the child, in five or six previous parturitions.

Dr. A. H. Goelet* has recently reported a case that could be labeled as one of "hour-glass contraction of the uterus before the expulsion of the fœtus." The woman was advanced to 6½ months of her third pregnancy. Labor came on, the os was fully dilated, the membranes were intact, no presenting part could be felt. The pains being feeble, ergot was given. After the rupture of the membranes and the discharge of a large quantity of amniotic fluid, "the uterus was discovered to be contracted in the center, the fœtus above the constriction, and a finger projecting through." Hemorrhage continued, and the indication to evacuate the uterus was urgent. "With great difficulty one finger was forced through the constriction, then another, and

* *North Carolina M. Jour.*, Mar., '78—Monthly Abstract.

after considerable search a foot was found, but having only two fingers with which to grasp it, very little traction could be used. Finally one foot (for the other could not be found) was dragged down and out of the vulva, but when the breech of the child presented against the constriction, it required all the force that could be exerted with both hands to bring it through, and the same was the case as the shoulders and head came down in succession."

I have been unable to consult the original report of this case, and regret the abstract is not more full and explicit in certain details. But judged strictly by the description given, the case is so similar to that of Dr. Gray that, from a pathological standpoint, but one very essential difference is to be noted; that, namely, in the period of gestation at which labor came on in the two cases. The inference is natural that the obstruction was anatomically alike in these cases.

Bearing upon this subject are the observations of Dr. Bandl.* He has recently studied 32 cases of rupture of the uterus, a majority of which occurred in the Vienna lying-in hospital, the rest (13) being from records of his own observation. In no case was this accident due to morbid change in the substance of the uterus. The ruptures nearly all occurred in or very near the cervix. In nineteen of the cases there was narrowing of the pelvis; in twelve there was either abnormal presentation or monstrosity of the child. Dr. B. believes the accident is due to excessive thinning and elongation of the cervix during labor, whereby it is predisposed to rupture, and that this thinning and elongation are due, to a large extent, to the "disproportion which does not allow the presenting part to descend into the pelvis." When this obstacle exists, the internal orifice of the cervix, contrary to the usual and normal course, "is raised a handbreadth above the brim" by the contracting effects of the womb, and perhaps by fixation of the external os against the brim of the pelvis, and so thinning of the walls and rupture of the cervix become possible. In some of the cases examined, the cervix was found eight inches in length.

---

*Centralblatt, No. 33, 1875. Amer. Jour. Med. Sci., Jan., 1876. Boston Med. & Sur. Jour., Jan. 3d, 1878.

It is a fact in favor of his hypothesis that of the six women whose histories are written by Drs. Hosmer, Kellogg and Gray, four had such a pelvic deformity as Bandl describes. Of the other two there is no record of any careful measurements—or measurements at all—to determine the question, although it is said simply that the pelves were normal. In one of these latter, it is certain, from the description, that a rupture of the uterus that occurred must have taken place through the cervical wall, warranting the suspicion that this was elongated and attenuated.

The preponderance among these cases of those with this deformity of the pelvis, not only tends to establish the identity and character of all of them, but further to confirm the hypothesis that the distocia in such cases is somehow connected with this particular kind and degree of deformity.

Whether the elongation of the cervix is the first step, and induces or favors the violent contraction of the internal os uteri, is an interesting question yet to be answered.

Very few writers on obstetrics make any reference to the fact that an hour-glass contraction may exist, the contracted portion consisting of the internal os and the two cavities respectively of the uterine body and cervix, and I am not aware of the existence of any treatise on the subject in which the possibility of an hour-glass contraction occurring before the birth of the fœtus is even remotely suggested.

Playfair * very distinctly departs from the old descriptions. He says the hour-glass contraction "seems to depend on spasmodic contraction of the internal os uteri, by means of which the placenta becomes encysted in the upper portion of the uterus which is relaxed."

Meadows † writes as though he expected somebody would challenge his assertions on this topic, for he says there was in all the cases he had seen (four) "a considerable space above the external os and the seat of the stricture, above which was encysted the placenta." He is satisfied "the space in question involved more than the cervical portion of the uterus—that, in fact, a good part of the body of the uterus was below the seat of stricture."

* Playfair's work on Midwifery, p. 376.

Roberts, * in his recent work, says: "When the contraction exists at the internal os, hour-glass contraction results, the uterus being divided into two compartments, with the placenta in the upper division." Then, as though unwilling to leave the impression that such is the only mode of production of this phenomenon, he says: "True hour-glass contraction is, however, of rare occurrence."

Meigs † says: "Hour-glass contraction depends either upon the contraction of the womb at the upper limit of the cervical portion, so that the after-birth is contained, as it were, in a separate cell, or the contraction may take place so as merely to include the placenta, still retaining its original connection with the uterus."

Bedford ‡ says, under the head of hour-glass contraction, that the constriction usually occurs "at the upper extremity of the cervix or in the body" of the uterus.

That the cervix may easily become elongated in parturition, there ought to be no doubt; perhaps such elongation as is described in the cases just quoted, ought hardly to occasion surprise. It must have occurred to every practitioner of experience to observe cases where, without any serious obstruction to normal delivery, the cervix was so much elongated as to become fixed between the child's head and the pubic arch.

A number of cases are on record in which the external os has been extruded beyond the vulva. Mr. Lever § has reported a case in which the elongated anterior lip of the os in a patient with a small pelvis was forced out of the vulva like a large polypus, and was not fully retracted until two days after the labor—the mass being treated with punctures to favor the evacuation of the fluid of the swelling, and with fomentations; the use of the catheter being required in the meantime for the evacuation of the bladder.

Dr. Chas. W. Earle, at the West Chicago Medical Society, October 14, related a case that occurred several years ago in his

---

* Guide to the Practice of Midwifery, p. 180.

† Edition of 1856, p. 446.

‡ Principles and Practice of Obstetrics, 1863.

§ Gry's Hospital Reports. First Series, volume vii.

practice, and that bears strongly on this point. The patient was a woman in her tenth labor.

There was retention of the placenta, and considerable hemorrhage, after the expulsion of the child. The hand was introduced to detach the after-birth, and on withdrawing it, the wrist was grasped by vigorous contraction of what was undoubtedly the internal os. This contraction was so strong that it offered no inconsiderable obstacle to the withdrawal of the hand. After the delivery of the child, there was discovered a mass of fleshy substance, hard and firm, projecting from the vulva, which proved to be a loop as large in diameter as the finger, and through which two or more fingers could be passed. The mass had not entirely disappeared twenty-four hours after the labor. It was subsequently retracted within the body, and the patient made a good recovery.

From Dr. Earle's description, this fleshy mass could have been nothing but the anterior lip of the external os of a cervix uteri remarkably elongated. Admitting this condition of things to have existed, it is not strange that the pinching of the attenuated cervix between the child's head and the os pubis, with the forcing of powerful propulsive uterine contractions, should have caused a rent that would make exactly the loop that was observed. Indeed, it appears to me a little remarkable that such ruptures near the free border of the cervix, when elongated so excessively, do not occur frequently. It is a fact of significance that with the elongation of the cervix in this case, there was also the vigorous contraction of the internal os. This is in favor of the notion that the internal os is disposed to contract with preternatural force from elongation of the cervix, as well as from excessive irritation of the part, which we know to have occurred in this instance.

Dr Earle's patient has since borne a child in a labor unattended with the discovery of anything peculiar about the neck of the womb.

At the same meeting, Dr. W. T. Belfield gave an account of a case that occurred at Cook County Hospital during his residence there, in which, after the delivery of the child, the border of the external os uteri projected from the vulva, and was mistaken by some of the internes for the border of the placenta which was retained. Efforts were made, by traction on the cord, to dislodge the placenta, but to no purpose. A further examination revealed the internal os some six inches above the vulva in a condition of firm contraction, grasping the cord, the placenta being imprisoned beyond and above it. The dilatation of the internal os, and the extraction of the placenta, was a tedious operation, but was accomplished without injury to the patient, who made a good recovery.

Dr. A. R. Barton* has reported a case of excessive elongation of the cervix uteri, which was an obstacle to delivery. The cervix had been elongated for several weeks prior to the confinement, to such a degree that it projected beyond the vulva and the os externum had become like cutaneous surface in its appear ance. When labor began, it protruded from the vulva four inches, and was eight inches in circumference. "The finger carried into the cavity of the tumor discovered nothing more than a continuous canal. Even when the tumor was carried up on the finger as high as possible, the child could not be reached. The fundus of the uterus was well up into the epigastric region."

The external os was so dense that dilatation was impossible, and ten incisions were made in its border before the child could be extracted. After the labor was over, the tumor was returned to the vagina, and remained. The woman recovered, but had prolapsus of the uterus after she had been about the house a few weeks. Two years later she became pregnant, carried the child to the full period, and was delivered in a normal labor.

Whether it shall be fully proven that the internal os uteri is the chief factor in the production of most of the cases of the hourglass contraction of authors, as well as of all such remarkable and anomalous cases of ante-partum obstructive contraction as the records quoted in this paper illustrate, remains to be seen. But that the internal os is involved in many more pathological states and phenomena than is generally supposed, seems evident. Indeed, the pathological possibilities of this part of the uterus appear to be only now receiving full attention. One of these,

* *The Medical Record*, Feby. 16, 1878, p. 129.

and one which we seem often to forget about, is an excessive irritability and hyperæsthesia—or rather hyperalgesia. Now that it is the general habit to treat cases of uterine disease locally, it is one of the most common observations to find the internal os the most sensitive part of the organ ; indeed often it is found the only part sensitive. The external os may be hooked with a tenaculum, slashed and harpooned with cutting instruments and the patient not be conscious of it, when the gentlest passing of the smallest sound through the internal orifice will cause her to start with pain.

It is to be expected that a state of excessive irritability of the internal os will be attended with contraction, spasmodic or otherwise, of its muscular fibers, or pain, or both, whenever it is subjected to irritation. That such is the case in both the impregnated and unimpregnated uterus, we have abundant evidence. With a high degree of irritability, an irritation otherwise trivial will lead to them, albeit perhaps with only moderate intensity. Excessive irritation may induce them when only a normal degree of irritability exists in the part. What ought we to expect—what not to expect, when with an internal os in the highest degree irritable and sensitive, a labor is attended with an injury to the mouth of the uterus that borders on mutilation ?

Hour-glass contraction occurs most frequently in cases, where during the labor, the orifice of the womb has been much irritated by instrumental manipulations. Such is the opinion of some writers on obstetrics.*

In contracted pelves the cervix is necessarily liable to unusual irritation and injury. This being true, spasmodic contraction of the internal os in such cases as are reported by Dr. Hosmer, ought not, perhaps, to be regarded matter for wonder. It is the degree and force of the constriction that is the fact unaccountable. Not even the supposition of a ring of cartilage explains this fully.

How it happens that at the time of labor a degree of irritability may exist that makes spasmodic and irregular contraction possible, seems difficult of explanation. But ought it to be ?

---

* See Byford's work on Obstetrics.

We know that women with uterine disease often become pregnant. So common an occurrence is it for ulceration and chronic inflammation of the cervix to exist during pregnancy that grave discussions have occurred among the profession as to the propriety of continuing local treatment of them during this period. The sensitiveness of the internal os just referred to as so common in gynæcological cases, and which is doubtless largely due to inflammation, is not a bar to conception. And it seems nothing more remarkable that this irritability should continue during gestation than that an ulcer should. If it persists in any case, it is of course present when labor occurs, whether this be at the full term or before; and being present at such time, it is in waiting to cause, on the slightest provocation, the most serious mischief. But the provocation is often severe in the shape of irritations and injury to the mouth of the womb; and it is quite possible that the physiological changes of gestation may, in certain patients and under certain circumstances, increase an irritability of the internal os that before conception was pathological, so that when labor occurs it is ten fold more formidable and troublesome than it would otherwise have been.

As the exalted irritability often exists, if it is capable of inducing the obstruction once, why may it not do so frequently, especially as irritation of the cervix in labor is any time liable to occur?

Taking all the considerations named into account, I question whether the explanation here suggested is not alone sufficient to account for all the cases of obstructive contraction of the internal os uteri in labor that ever occurred.

The following history of a case,* reported as one of "stricture of the internal os as a cause of miscarriage," by Dr. Wm. Marshall, is quoted here because it exemplifies the most extreme hyperæsthesia and propensity to spasm. The patient was a delicate woman of thirty, and five months advanced in pregnancy. Her last previous pregnancy had resulted in miscarriage at the fifth month, with pains more severe than was usual in her con-

---

* *Glasgow Medical Journal*, Feb. 1869—*Am. Journal.*

finements at term. The os was dilated "to the size of half a crown, and very soft."

"On passing my finger up in order to feel the fœtus, I found the canal of the cervix becoming decidedly narrower, when suddenly she cried out that I was cutting her, and jerked herself away. On a second attempt, the same thing was repeated; but on a third, being prepared for her moving, I ascertained that a tight resisting constriction existed at the internal os, which would not admit the tip of the finger. As soon as I touched the constricted part, she complained of a severe cutting pain; and on attempting to pass the finger through it, she became hysterical, and on my persisting, *perfectly maniacal.* On withdrawing my finger, she immediately became rational, and complained of the agonizing pain I had caused her.

"As she was quite positive that in her previous miscarriage she had suffered for three hours as much as she was doing now, I waited for a couple of hours. During this time the pains were very strong, and the suffering greater than I had ever seen in any confinement. In order to make a thorough examination, I put her under chloroform. The external os was very soft and dilated; but at the internal os there existed a constriction, which still readily allowed the finger to pass through, and which seemed now quite dilatable. The breech was presenting, and I had no doubt that when a pain came it would be pushed through, and the whole thing soon be at an end. The pains, however, did not return as long as I left her under chloroform, so that I was forced to discontinue it. The stricture returned with the first pain, firmly grasping the tip of my finger, which I had retained in the uterus. I now gave her a dose of ergot, and waited until one o'clock, when, finding that little or no progress had been made, I determined to notch the stricture in one or two places under chloroform, as it was impossible to touch it without causing intense pain, and bringing on a maniacal paroxysm. I went home for a probe pointed bistoury, and on my return in half an hour, found the strictured part with the breech forced into it, protruding through the internal os, which was drawn up around it. After a few pains, the breech passed through the constriction. I pulled down the body, and finding that the head would not come, pushed my finger past it, hooked it over the crown and pulled the head through the stricture. Without withdrawing my finger, I detached the placenta, and withdrew it and the finger at the same time. While doing all this, the patient was perfectly maniacal—she shrieked, kicked, struck, and bit at those around her."

"She recovered without a bad symptom."

This case is a most interesting one in several particulars. In some points it will be seen to resemble the case, the report of which introduces this paper. It was attended with a much greater degree of sensitiveness and irritability of the internal os uteri, more pain and probably more vigorous spasmodic contraction, but there was no such elongation of the cervix, and distension of its body into a large cavity—a cavity capable of accommodating a fœtus, as existed in that case.

That it was the internal os alone that was the offending part and the cause of the obstruction in this case is evidenced by Dr. M's. description of his exploration of the external os, the cervix—which grew narrower toward its upper extremity, and the strictured point itself. If confirmation were needed, it is furnished by the phenomena of the expulsion of the presenting part of the fœtus from the womb. "The strictured part, with the breech forced into it," protruded "through the external os, which was drawn up around it," the cervix being literally turned inside out. This case is, fortunately, reported with such explicitness and detail that it alone establishes the pathological conditions described.

In the case reported by myself there was a total absence of hyperæsthesia of any part of the womb. The vigorous contraction of the internal os was probably in part due to an increase of its normal physiological irritability—its capacity to contract in response to stimuli. But the main cause, it seems to me, was the irritation produced by the stretching of the cervix, aided possibly by a pathological condition of the external os—traumatic or otherwise—evidence of which in a distinct roughness of its surface, was discovered at the first examination.

The distension of the cervix was probably a purely mechanical process. The blood oozed into it from the cavity of the uterus; retarded by the closure of the external os, coagulation took place; the accretion continued; and thus, slowly, the enlargement came on. Finally the limit of the capacity of the part was reached and the external os gave way in dilatation.

